# Evaluation of antioxidant and anti-inflammatory potential and *in silico* tyrosinase binding interactions of edaravone derivatives

**DOI:** 10.1080/14756366.2025.2561678

**Published:** 2025-10-10

**Authors:** Naveen V. Kulkarni, Anaswara S. A., Induja S. A., Dineshchakravarthy Senthurpandi, Dimitar G. Bojilov, Stanimir P. Manolov, Iliyan I. Ivanov, Jamelah S. Al-Otaibi, Y. Sheena Mary

**Affiliations:** ^a^ Department of Chemistry, Amrita Vishwa Vidyapeetham, Amritapuri, Kerala, India; ^b^ Center of Molecular and Macromolecular Studies, Polish Academy of Sciences, Lodz, Poland; ^c^ Department of Organic Chemistry, University of Plovdiv, Plovdiv, Bulgaria; ^d^ Department of Chemistry, College of Science, Princess Nourah bint Abdulrahman University, Riyadh, Saudi Arabia; ^e^ Department of Physics, FMNC, University of Kerala, Kollam, India

**Keywords:** Edaravone, antioxidants, anti-inflammatory compounds, tyrosinase binding, MD simulation

## Abstract

Two edaravone derivatives were synthesised and characterised by using several spectral and analytical techniques. The antioxidant activities of these organic compounds were analysed by using HPSA, DPPH and ABTS·+ assays. Anti-inflammatory property of the synthesised derivatives was analysed by evaluating albumin denaturation inhibition abilities. Optical energy band gaps were evaluated using the Tauc plots. Computational method was used to analyse the frontier molecular orbitals of the compounds and MEP surface analysis was used to identify the nucleophilic and electrophilic attacking sites. Owing to the higher antioxidant potential the interaction of the compound **2** with the protein Tyrosinase (isolated from the bacterium, *Bacillus megaterium*) was investigated using detailed molecular docking and simulation methods. Compound **2** exhibited higher free radical scavenging activity, good anti-inflammatory property and found to effectively bind to the Tyrosinase protein. These derivatives have potential application in the production of improved antioxidant and anti-inflammatory agents as well as cosmeceuticals.

## Introduction

Antioxidant molecules play an important role in controlling reactive oxygen species (ROS) & reactive nitrogen species (RNS) and prevent the possible oxidative damage on key biomolecules such as DNA, proteins, and lipids^1–5^. Owing to their unique protective properties, antioxidant compounds hold significant importance in both pharmaceutical and cosmeceutical applications^6–9^. In pharmaceutical research, antioxidants are well explored as therapeutic agents to control oxidative-stress related diseases, ranging from neurodegenerative disorders to cardiovascular disorders^10–12^ while in cosmeceutical applications, they are known for preventing skin ageing, and reducing inflammation^13–15^. The antioxidant compounds such as polyphenols, vitamins C and E, resveratrol, coenzyme Q10, curcumin etc., are obtained from natural sources^16–18^, while several others like butylated hydroxyanisole (BHA), butylated hydroxytoluene (BHT), propyl gallate and derivatives of indole, pyrazine, quinoline etc., are synthesised in the lab^19–25^. Natural antioxidants have the advantage of high biocompatibility, broader biological impacts, and sustainable nature, even though they suffer from inconsistent potency and lower stability^4^^,^^26–29^. On the other hand, synthetic antioxidants offer long shelf life, dependable performance, and economic scalable production, but have concerns about side-effects and environmental impact^4^^,^^30–33^. Therefore, it is important to consider all these aspects while developing potential antioxidant molecules for the effective therapeutic and cosmetic applications.

**Figure 1.**
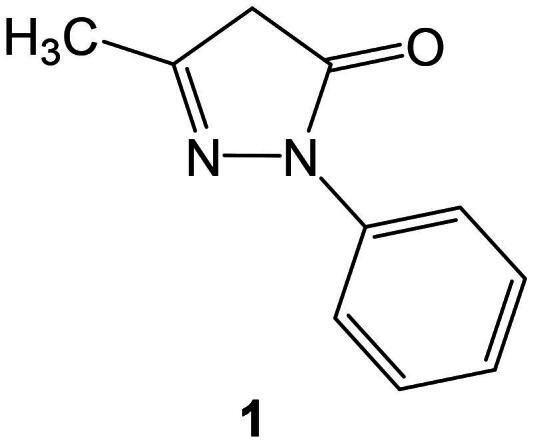
Molecular structure of edaravone.

Edaravone (**1**) (Figure 1) is known for its radical scavenging activity and hence been explored in the varieties of bioapplications, such as antioxidant, antibacterial, anti-inflammatory, and anticancer applications^34–41^. Edaravone is proposed to follow three different mechanisms to work against oxidative stress. It is shown to prevent the peroxyl radical-induced peroxidation systems in both aqueous and lipid media, to stop lipoxygenase and non-enzymatic peroxidation of lipids, and to inhibit both OH-dependent and OH-independent peroxidation of lipids^42^.

It is established that, the antioxidant efficacy of edaravone is to closely connected to the electron density of the pyrazolone ring, as it plays a key role in facilitating electron transfer mechanisms responsible free radical scavenging process^43^. Modifying the molecular structure of edaravone by introducing substituents with suitable stereo-electronic characteristics could enhance its antioxidant capacity as well as help in improving lipophilicity, hence enhance and expand its pharmaceutical applications^34–44^. Many strategic routes have been employed to modify the molecular structure of the edaravone to improve its potency. The most common routes are substitution on the N-phenyl moiety, derivatization of the ketonic group at the 5-position, modification of the methyl group at the 3-position, and substitution at the 4-position of the pyrazole ring^45–53^. These derivatization strategies have demonstrated considerable success in modulating the biological activity of edaravone analogs. Among them, substitutions at the 4-position of the pyrazole ring have shown particular promise, yielding derivatives with significantly improved lipophilicity and potent antioxidant properties^34^^,^^41^^,^^54^^,^^55^.

In our previous work we have reported the evaluation of antioxidant activity and melanogenic inhibitory activity of eight derivatives of edaravone. It was established that, the substitutions at the 4-position of the pyrazole ring plays an important role in deciding the antioxidant activity of the compounds. Among the eight derivatives, the compound containing a semicarbazone substituent at the 4-position of edaravone was found to exhibit better antioxidant activity and a strong interaction with Tyrosinase protein demonstrating its potential melanogenic inhibitory action^41^. In the current work, we are reporting the synthesis and evaluation of the antioxidant activity of the two new derivatives of the edaravone. The first derivative was obtained by reacting edaravone with benzaldehyde and the second derivative was obtained by reacting the first derivative with piperidine. Both the derivatives are thoroughly characterised using spectral and analytical techniques and evaluated for their antioxidant efficacy using three experimental radical scavenging analysis methods.

It is documented that, being excellent antioxidant agents, the edaravone derivatives can also play important role in reducing inflammation. They effectively control the oxidative stress and neutralise the free radicals and hence support the anti-inflammatory mechanism^56–60^. Accordingly, the anti-inflammatory properties of the synthesised edaravone compounds were investigated using albumin denaturation inhibition method and the results are discussed in this report.

Further the molecular structures of both edaravone derivatives were established with the help of DFT calculations. The frontier molecular orbital energy levels of the molecules and their chemical characteristics were determined. It is known that Tyrosinase enzyme plays key role in the process of melanogenesis; inhibition of activity of tyrosinase enzyme via binding interactions is a desired property for a potential antioxidant and depigmenting agents^61–64^. Consequently, the binding interaction of our best performing antioxidant agent with the tyrosinase protein (3NM8) was analysed, in detail, using molecular docking and molecular dynamics simulation studies. The outcomes are presented in this report.

## Experimental

### Materials and methods

All the starting reagents and chemicals used in synthesis and analyses were purchased from Sigma-Aldrich, Sd-Fine and Spectrochem. Water used in the analysis was purified by Millipore purifier (Millipore, St. Louis, MO, USA). Human albumin 20% – BB, 200 g/L was purchased from BB-NCIPD Ltd., Bulgaria. IR spectra were recorded on Perkin Elmer FTIR instrument in ATR mode (range 4000 − 500 cm^−1^). The UV-Visible spectra were recorded on Perkin Elmer lamda-25 and SHIMADZU-1800 UV-VIS spectrophotometer. NMR analysis was carried out on a Burker 400 Hz instrument. Single crystal X-ray analysis was carried out using a Bruker APEX-II Kappa machine. Camspec M508 spectrometer was used in the bio-activity assays.

### Synthesis

Edaravone (**1**) was obtained from Sigma-Aldrich and recrystallized from methanol before use.

#### 4-benzylidene-5-methyl-2-phenyl-2,4-dihydro-3H-pyrazol-3-one (compound 2)

**Figure 2.**
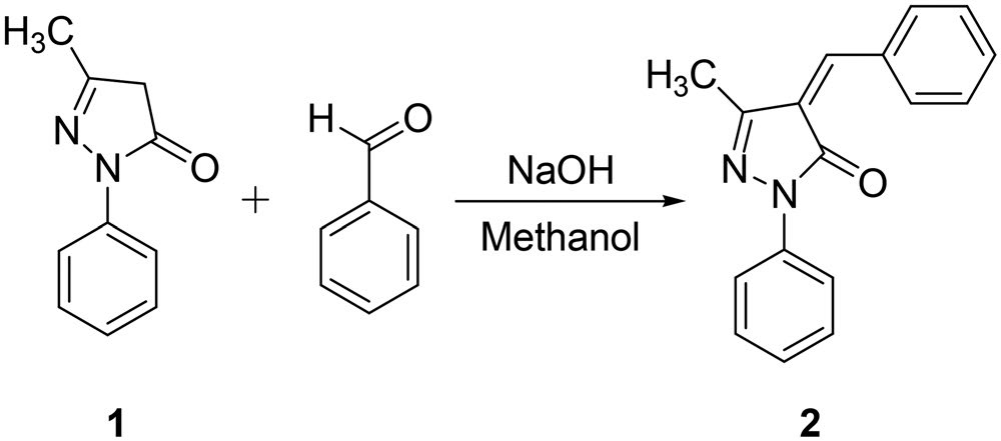
Synthetic route of compound **2**.

Compound **2** was prepared by following the procedure described below. 1.73 g of edaravone was weighed and transferred into an RB flask. A mixture of 5 g NaOH in about 30–40 ml methanol was prepared, then transferred to the RB containing edaravone. The resulting mixture was of cream colour and was left for stirring for 10 min. To this mixture, about 1.015 ml of benzaldehyde was added upon which the solution turned orange. This resulting mixture was left for stirring for 12 h. After 12 h, the mixture was neutralised with dilute HCl (until pH 7) to precipitate the product. The product was allowed to settle down and was filtered followed by drying. The product was purified by passing through a silica plug, eluted with ethyl acetate: hexane mixture (1:1). The recrystallization of the compound was done in methanol (Figure 2) (Yield − 92%)^65^^,^^66^.

#### 5-methyl-2,4-diphenyl-4-(piperidin-1-yl)-2,4-dihydro-3H-pyrazol-3-one (compound 3)

**Figure 3.**
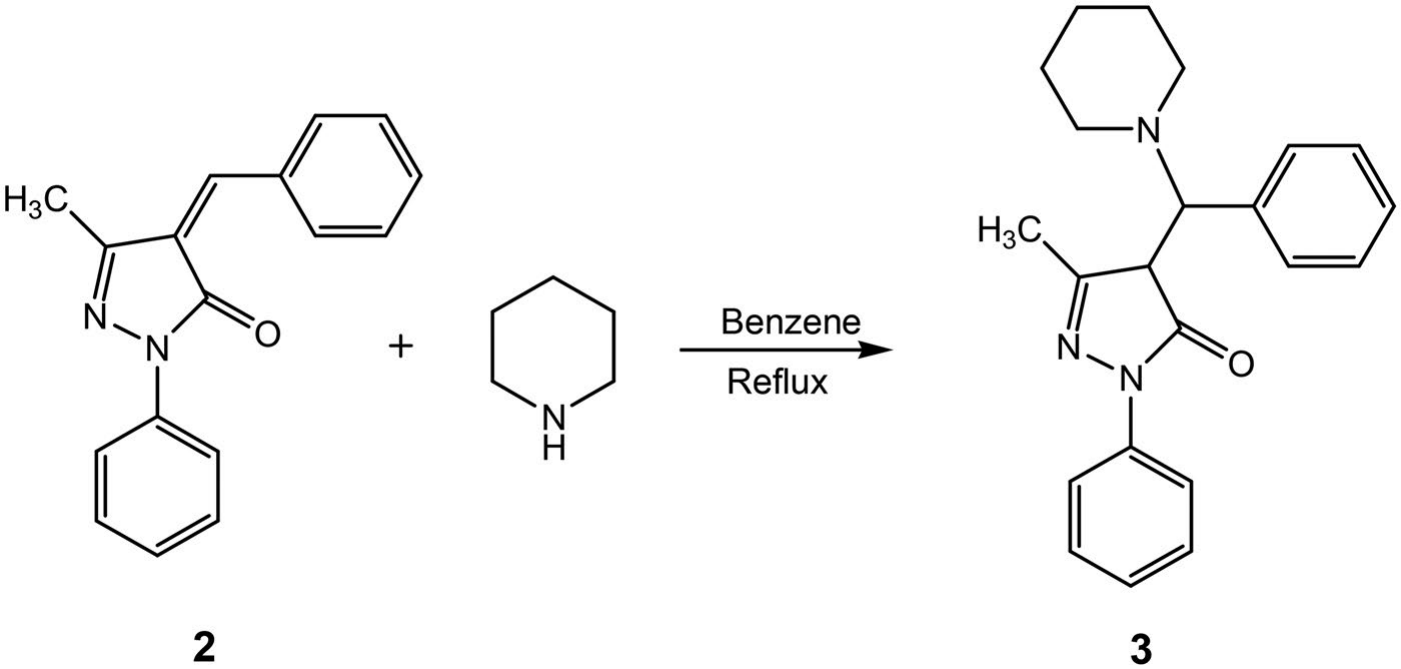
Synthetic route of compound **3**.

Compound **3** was prepared by following the procedure described below. 4-benzylidene-5-methyl-2-phenyl-2,4-dihydro-3H-pyrazol-3-one was weighed and transferred into an RB flask containing 30 ml benzene. To this mixture 0.104 ml piperidine was added using a syringe. The resulting mixture was left for reflux for 12 h. The product formed after 12 h was then precipitated by adding hexane. The product was purified by passing through silica column using ethyl acetate:hexane (1:1) eluent. The product was recrystallized using methanol (Figure 3) (yield − 86%)^67^^,^^68^.

### Analysis of antioxidant activity of edaravone derivatives

Analysis of antioxidant activity of the synthesised edaravone derivatives was done by using three different methods, Hydrogen peroxide scavenging activity (HPSA), 1,1-diphenyl-2-picrylhydrazyl (DPPH) and 2–2′-azino-bis-(3-ethylbenzothiazoline-6-sulfonate) (ABTS·+) assay^41^.

### Hydrogen peroxide scavenging assay (HPSA)

The method developed by Manolov et al. was used to evaluate the free radical scavenging capacity of edaravone derivatives^69^. The potassium phosphate buffer solution (0.2 M, pH 7.4) was used to prepare the hydrogen peroxide solution (43 mM). The sample was analysed as follows: 0.6 ml of H_2_O_2_ (43 mM), 1 ml of a sample with varying concentrations (10–1000 μg/mL), and 2.4 ml of buffer solution were mixed in test tubes. After agitating the mixture, it was left in the dark at 37 °C for 10 min. Using a spectrophotometer, absorbance was measured at 230 nm in relation to a blank solution that contained hydrogen peroxide and phosphate buffer but did not contain the sample. The standard used was ascorbic acid. The following formula was used to determine the percentage HPSA of the sample, which was assessed by comparing them to a blank sample:

$$I,\text{ \%}\left(\text{HPSA}\right)=\left[\frac{A_{\text{blank}}-\left(A_{\text{TS}}-A_{\text{CS}}\right)}{A_{\text{blank}}}\right]\times 100$$

Where A_CS_ is the absorbance of the control sample (test sample + phosphate buffer), A_TS_ is the absorbance of the test sample (test sample + phosphate buffer + hydrogen peroxide), and A_blank_ is the absorbance of the blank sample (phosphate buffer and hydrogen peroxide). Through the process of interpolating the graphical dependence of scavenging hydrogen peroxide on concentration, the mean IC50 value was calculated using three replicates.

### Free radical scavenging assay

The Docheva et al. method^70^ was used to evaluate the DPPH• technique. A 0.12 mM concentration of DPPH• reagent solution was made every day. 2 ml of the test samples (various concentrations) were added to a vessel containing 2 ml of the DPPH• solution. The resulting mixture was kept at room temperature for 30 min in the dark. A spectrophotometer (Camspec M508, England) was used to measure the absorbance of the sample at 517 nm. All the measurements were carried out under dim light. The following formula was used to calculate the percentage RSA of the sample, which was assessed by comparing them to a control:

$$\text{RSA},\text{\%}=\left[\frac{A_{0}-A_{b}}{A_{0}}\right]\times 100$$

where A_0_ represents the absorbance of the control blank and A_b_ represents the absorbance of the samples. Using three trials as the basis, the mean IC50 was determined by interpolating the graphical dependency of the concentration and degree of DPPH radical inhibition.

### ABTS assay

The colorimetric approach described by Re^71^ was slightly modified to measure the antioxidant capacity. In order to prepare the 2,2′-azino-bis(3-ethylbenzothiazoline-6-sulfonic acid) cation radical (ABTS•+) solution for this experiment, 7 mM of ABTS was dissolved in 2.45 mM K_2_S_2_O_8_. This mixture was mixed in the dark for 12 to 16 h at room temperature to achieve a stable oxidative state. The ABTS•+ stock solution was diluted with methanol for the extract analysis until the absorbance at 734 nm became 0.70 ± 0.02. This is how the sample analysis was carried out: 2.85 ml of the ABTS solution was combined with 0.15 ml of the sample. The samples were incubated for 7 min at room temperature before their absorbance was measured at 734 nm using a spectrophotometer (Camspec M508, England). The outcomes were expressed as the IC_50_ mentioned previously.

### Inhibition of albumin denaturation (IAD) analysis

*In vitro* analysis of anti-inflammatory activity of compounds **2** and **3** was assessed as inhibition offered of the compounds towards denaturation of the protein albumin. The analysis was performed according to the reported Manolov method with a minor modification^72^. The experiment was performed with human albumin. The solution of albumin (1%) was prepared in distilled water (pH 7.4). Test samples/standards were first dissolved in 1 ml of DMSO and then supplemented with 1% Tween 80 in PBS so that the final concentration of the stock solution was 1000 μg/mL. Then a series of working solutions with different concentrations (20–500 μg/mL) in 1% Tween 80/PBS were prepared. The reaction mixture containing 2 ml test sample/standard and 1 ml of 1% albumin solution. It was then incubated at 37 °C (15 min) and then heated to 70 °C (15 min). Turbidity of the solution was measured using a spectrophotometer (at 600 nm). Ibuprofen was used as standard. The experiments were done in triplicate. Percentage inhibition was calculated against control (plain albumin solution).

$$\text{\% }\text{IAD}=\left[\frac{A_{\text{blank}}-A_{\text{sample}}}{A_{\text{blank}}}\right]\times 100$$

## Statistical analysis

All experiments were conducted in triplicate. Data are presented as mean ± standard deviation (SD). Statistical significance was defined at *p* < 0.05. Data analysis was performed using SPSS version 19.0 (SPSS Inc., Chicago, IL, USA). One-way analysis of variance (ANOVA) was employed, followed by Duncan’s *post hoc* test to assess differences between mean activity values.

### Computational analysis

The molecular structures of the compounds were determined by Density Functional Theory using Gaussian 16 and Gauss view software^73^^,^^74^. For all the calculations B3LYP technique with the 6–311++G* basis set was utilised^75^. In the docking studies, in order to have a proper docking validation and efficiency of ligands binding were compared with glyphosate as standard, using Autodock vs 4.2.6 to perform docking investigations on the molecule in question^76^. Both receptor and target compound were then saved in pdbqt format after combining non-polar hydrogens. Molecular docking was performed within a grid box dimension 25 × 25 × 25 Å with 0.3 Å spacing. Docking experiments were carried out as per the Lamarckian Genetic Algorithm (LGA) in triplicates. Each of investigations (in a default setting) included 50 solutions, a population size of 500, 2500000 evaluations, a maximum generational number of 27. After the completion of docking, the RMSD clustering maps were generated with the clustering tolerances of 0.25, 0.50, and 1 to find the best cluster that had the lowest energy score and the most populations. MD simulations of the protein 3NM8 in conjunction with compound **2** was executed using vs 2020.1 Desmond simulation engine^77^^,^^78^.

## Results and discussion

### Physical and chemical properties

Both the synthesised compounds **2** (yield − 92%) and **3** (yield − 86%) are found to be colourless solids with crystalline nature. Both are soluble in ethanol, chloroform, dichloromethane, acetonitrile and DMSO but are insoluble in water. Compound **2** and **3** melt in the range 194–196 °C and 218–220 °C, respectively.

### Elemental analysis

The experimentally determined C, H and N content of the compounds **2** & **3** are in well agreement with the expected molecular structures, thus confirming their analytical purity. The values are provided in Table 1.

**Table 1.** Elemental analysis data of the compounds (average values of triplicate measurements).

Compound	Empirical formula	C%, found (calculated)	H%, found (calculated)	N%, found (calculated)
**2**	C_17_H_14_N_2_O	77.46 (77.84)	5.62 (5.38)	10.97 (10.68)
**3**	C_22_H_25_N_3_O	75.88 (76.05)	7.16 (7.25)	12.32 (12.09)

### FTIR analysis

The infra-red studies of both the organic compounds were carried out in the form of KBr disc. In the IR spectrum of compound **2**, bands at 2949 and 2851 cm^−1^ corresponds to C–H stretching of the aryl and alkane groups, band at 1638 cm^−1^ corresponds to carbonyl group (C = O) and 1591 cm^−1^ corresponds to the C = C stretching. The C = N band is obtained at 1492 cm^−1^ and signals at 1168 cm^−1^ corresponds to the C–N stretching. On the other hand, in the IR spectrum of compound **3,** the bands corresponding to C–H stretching of aryl and alkane groups have shifted to 3060 and 3029 cm^−1^. Signals at 2938, 2857 and 2532 cm^−1^ corresponds to the C–H stretching in the piperidine ring. A medium intensity band is obtained at 1620 cm^−1^, which is due to the carbonyl group (C = O). The C = N signal is obtained at 1498 cm^−1^ and the band at 1168 cm^−1^ corresponds to the C–N stretching^79–85^.

### UV-Vis spectral analysis

**Figure 4.**
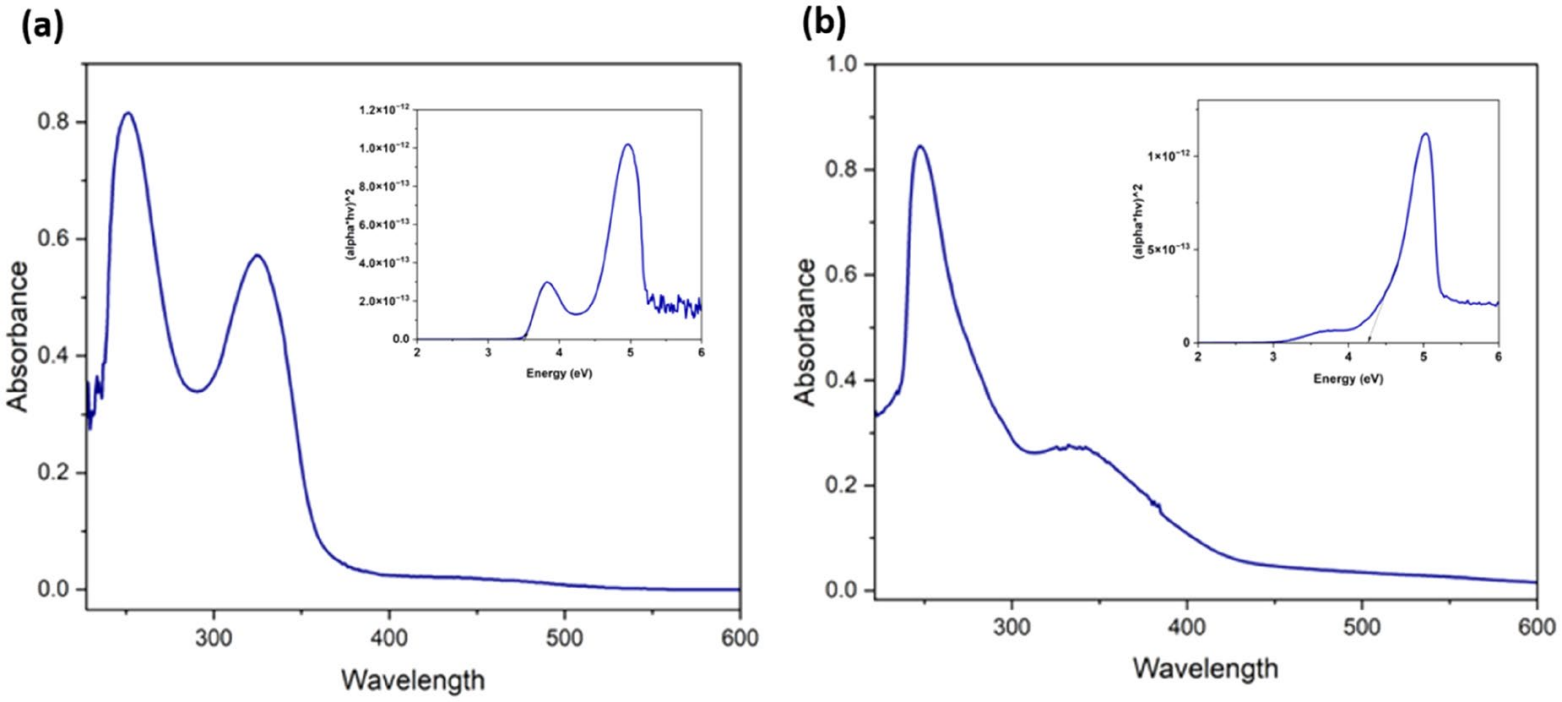
The UV-visible absorption spectra of compounds **2**(a) and **3**(b). Inner images are the corresponding Tauc plots used to determine the direct optical band gap values of the compounds.

UV analysis of both the organic compounds **2** and **3** was carried out in a methanol solution at 1 0 –3 M concentration (Figure 4). In the electronic spectrum of compound **2**, the peaks at 251 nm and 325 nm corresponds to n–π* transition and π–π* transition respectively. In the case of compound **3**, the peaks at 248 nm and 340 nm are attributed to n–π* transition and π–π* transitions^79–85^.

### Tauc plot: calculation of optical band gap values

Using the UV-visible spectral data and Tauc plot method, optical band gap values of both the compounds are calculated (Figure 4)^84^^,^^85^. The direct band-gap values of both the compounds are listed in Table 2. The compound **2** has a lower direct optical band gap value as compared to the compound **3**.

**Table 2.** Optical band gap (direct) values of the synthesised compounds.

Compound	Optical band gap (direct)
**2**	3.52 eV
**3**	4.26 eV

### ^1^H NMR analysis

^1^H NMR studies of the compounds **2** and **3** was done in DMSO-d6. Both the compounds showed relatively broad peaks. In the ^1^H NMR spectrum of compound **2**, a singlet peak at δ 1.30 ppm is assigned to the CH_3_ group protons. Single peak at *δ* 7.20 is assigned to olefinic proton. A broad multiplet peak at *δ* 7.40–7.50 ppm corresponding to 5 protons is ascribed to the aromatic protons of the phenyl ring directly connected to the pyrazolone nitrogen. Peaks observed at δ 7.12 ppm (1H), 7.30 ppm (2H) and 7.65 ppm (2H) are attributed the aromatic para, meta and ortho protons of the phenylethene fragment. Similarly, in the H^1^ NMR spectrum of compound **3**, broad multiplet observed at *δ* 1.54, 1.61, and 2.97 ppm are ascribed to the protons in the piperidine ring. Singlet peak observed at *δ* 2.15 and ppm is due to CH_3_ protons. Singlet peak observed at *δ* 4.61 is assigned to the C–H proton of benzyl group. A set of triplet (1H), triplet (2H) and doublet (2H) observed at *δ* 7.04, 7.18 and 7.39 are assigned to the aromatic protons of the benzyl group. The other three peaks observed at *δ* 7.03 (triplet, 1H), 7.30 (triplet, 2H) and 7.94 (doublet, 2H) are assigned to the para, meta and ortho-protons of N-phenyl ring^41^^,^^65–68^^,^^79–83^.

### Single crystal X-ray analysis

**Figure 5.**
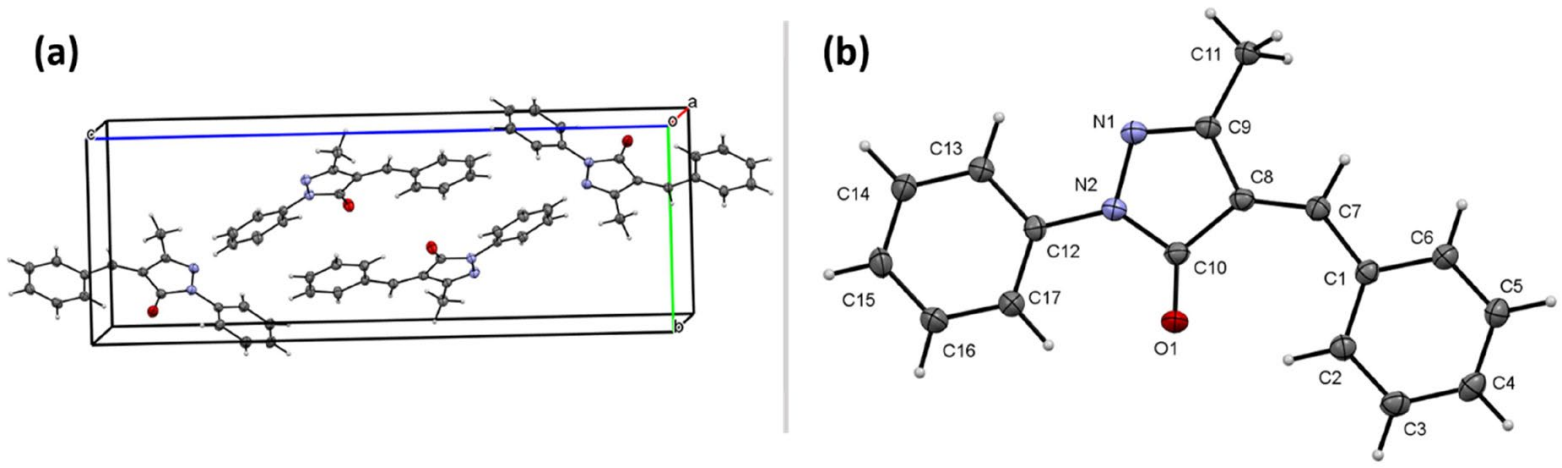
(a) Monoclinic unit cell of the compound **2** showing four molecules (a = 6.0544(6) Å, b = 8.8927(9) Å, c = 24.979(3) Å, *β* = 96.507(4)°, V = 1336.2(2) Å^3^); (b) Molecular structure of compound **2** (50% probability ellipsoids). Selected bong lengths, O1-C10 = 1.222(2), N1-N2 = 1.399(2), N1-C9 = 1.303(2), N2-C10 = 1.388(2), N2-C12 = 1.415(2), C1-C7 = 1.460(3), C7-C8 = 1.349(2) Å; Selected bond angles, C9-N1-N2 = 106.77(14), N1-N2-C12 = 117.62(14), C10-N2-N1 = 112.49(15), C10-N2-C12 = 129.36(15)°.

Compound **2** was crystalised from a methanolic solution layered with hexane to obtain X-ray quality crystals. The crystal obtained was found to have a monoclinic unit ell with space group P21/n containing four molecules of compound **2** (CCDC deposition no. 2448826). The unit cell and molecular structure of the compound **2** is represented in Figure 5 and key bond length and bond angles are listed. The crystal parameters are provided in Supplementary Information.

### ESI-mass analysis

As suitable crystals of compound **3** could not be obtained for X-ray analysis, ESI mass analysis was used to further confirm the molecular structure of the compound. The highest intensity peak observed at *m/z* 347 in the corresponding mass spectrum (+ve scan mode, see Supplementary Material) is assigned to the molecular ion [M]^+^, confirming the molecular structure of the molecule. The corresponding [M + 1]^+^ peak is observed at *m/z* 348 as a lower intensity peak^65^^,^^66^.

### Analysis of antioxidant activity

Reactive oxygen species (ROS) comprise chemically reactive oxygen-derived radicals and molecules, including superoxide (O_2_^•-^), hydroxyl (^•^OH), peroxyl (ROO^•^), and alkoxyl (RO^•^) radicals, as well as hypochlorous acid (HOCl), ozone (O_3_), peroxynitrite (ONOO^-^), singlet oxygen (^1^O_2_), and hydrogen peroxide (H_2_O_2_). They are naturally generated as byproducts of cellular metabolism. Under physiological conditions, enzymatic systems regulate ROS levels. It is well established that ROS can damage vital biomolecules of considerable biological importance, including phospholipids, proteins, and DNA. Such oxidative damage has been demonstrated to contribute to the pathogenesis of numerous diseases, including cancer, cardiovascular disorders, atherosclerosis, and Alzheimer’s disease^86^. The accumulation of ROS within the organism has been associated with a reduction in lifespan^87^. The generation of ROS is further accelerated in the presence of inflammatory processes. Consequently, the present study focuses on the deactivation of hydrogen peroxide and the assessment of radical-scavenging activity, employing the DPPH and ABTS methods. Hydrogen peroxide is an oxidising agent that is continuously produced in living tissues as a result of several metabolic processes. However, its detoxification is crucial to prevent its involvement in harmful reactions, such as the Fenton reaction. In this reaction, H_2_O_2_ interacts with ferrous ions (Fe^2+^), generating highly reactive and deleterious hydroxyl radicals (•OH)^88^. The obtained results regarding the antioxidant activity of the synthetic edaravone analogues were subsequently compared with that of natural edaravone, which served as the reference standard.

The antioxidant activity of the compounds was examined using HPSA, DPPH and ABTS^69–71^. The below data contain the results of edaravone (**1**) and synthesised derivatives **2** and **3**. Three set of each analysis were performed. The data was reported as mean ± SD. A significant threshold of *p* < 0.05 was app0lied. The outcomes are presented as IC_50_ ± SD, µg. mL^−1^. Ascorbic acid (IC_50_ = 24.84 µg. mL^−1^) was used as a standard. The analysis data is provided in Table 3. In the HPSA assay, IC_50_ values of **2** and **3** are found to be significantly lower than that of the edaravone **(1)**, indicating their higher antioxidant potential. Among the two derivatives, compound **3** is found to be relatively more active than compound **2**. In the DPPH assay, both the compounds **2** and **3** exhibited almost equal IC_50_ values and their antioxidant potential was found to be comparable to edaravone (1). In the ABTS assay, both the compounds exhibited relatively lower antioxidant activity as compared to the parent molecule, edaravone (**1**).

**Table 3.** Antioxidant activity results (HPSA, DPPH, ABTS) of edaravone (**1**) and its derivatives **2** and **3** (The IC50 values of edaravone **1** are taken from our previous work^41^ for the comparison, since same experimental setup is used in the current study as well).

Compounds	IC_50_ ± SD/μg. mL^–1^		
	HPSA	DPPH	ABTS
1*	100.56 ± 10.51	14.94 ± 0.31	51.88 ± 0.54
**2**	81.86 ± 6.54	13.71 ± 0.35	133.53 ± 4.09
**3**	78.94 ± 1.05	13.88 ± 0.42	125.74 ± 1.54

### Inhibition of albumin denaturation (IAD) studies

Anti-inflammatory activity is commonly evaluated using various biochemical assays, including the Inhibition of Albumin Denaturation (IAD) method. The IAD assay enables the assessment of compounds’ ability to suppress inflammatory processes by inhibiting specific enzymes or signalling pathways involved in inflammation. This evaluation is critical for the identification of potential therapeutic agents with anti-inflammatory properties and for the development of treatments targeting chronic inflammatory diseases. The inhibition percentages of the two synthesised edaravone derivatives **2** and **3** are presented in Table 4. The results are expressed as IC_50_ values. Ibuprofen, a well-established anti-inflammatory agent, was used as a reference standard for comparative evaluation of the activity of the newly synthesised edaravone derivatives. The IC_50_ values of 107.25 ± 1.30 and 106.20 ± 2.64 indicate a potential stabilisation of protein structures and the possible inhibition of inflammatory cascades involving cyclooxygenase (COX) or lipoxygenase (LOX) enzymes. However, their inhibitory potential was found to be slightly inferior to the standard, ibuprofen (IC50 = 76.05 µg/mL)^68^^,^^72^.

**Table 4.** Results of the IAD of edaravone derivatives and standard Ibuprofen presented as IC_50_, µg/mL.

Sl. No.	Sample	IAD analysis *IC_50_, µg/mL*
1	2	107.25 ± 1.30
2	3	106.20 ± 2.64
3	Ibuprofen	76.05 ± 1.04

### Computational analysis

**Figure 6.**
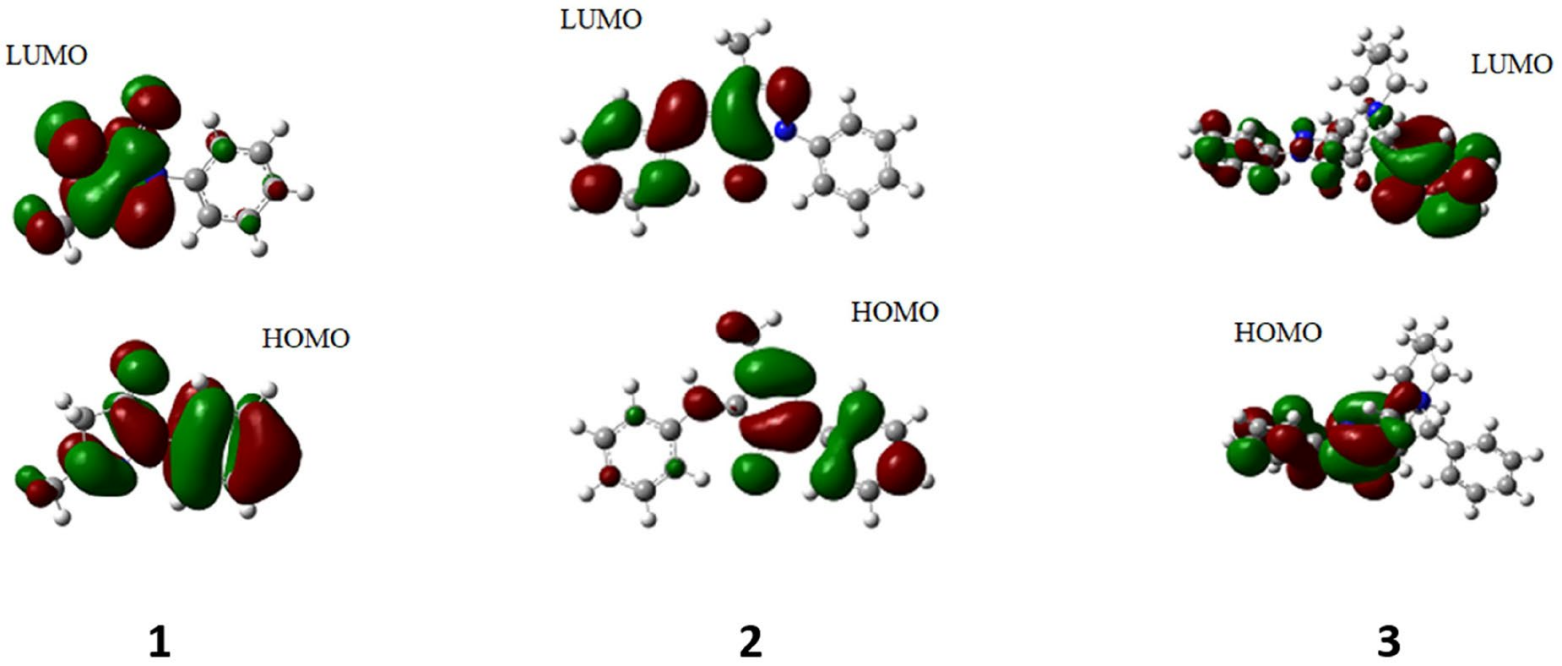
Molecular structures depicting the frontier molecular orbitals of edaravone **1** and derivatives **2** and **3**.

The molecular structures of the edaravone **1** and derivatives **2** and **3** were derived. The molecular structures of the compounds with highlighted frontier molecular orbitals are represented in Figure 6. From the DFT analysis, it is seen that (Table 5), the band gap value of compound **2** is lower (i.e. 3.0038 eV) as compared to edaravone **1** (i.e. 4.7611 eV) and compound **3** (i.e. 4.8490 eV). This is in line with the optical band gap values obtained from the Tauc plots (*vide supra*). The low band gap value indicates that, the compound **2** has better antioxidant properties as compared to the edaravone **1** and compound **3**^41^.

**Table 5.** The electrophilicity indices values of the compounds obtained by DFT calculations (Values for edaravone **1** are taken from previous work^41^).

Compounds	Energy of HOMO	Energy of LUMO	Gap	Hardness	Chemical potential	Electrophilicity index
**1**	−5.8392	−1.0781	4.7611	2.3806	−3.4587	2.5124
**2**	−5.5521	−2.5483	3.0038	1.5019	−4.0502	5.4611
**3**	−5.7230	−0.8740	4.8490	2.4245	−3.2985	2.2438

The other parameters obtained from the DFT calculation, such as hardness, chemical potential and electrophilicity index (Table 5) also indicate the higher reactivity of the compound **2**. While compound **3** is found to be relatively less reactive than the parent molecule edaravone **1** itself.

### Molecular electrostatic potential surface analysis

**Figure 7.**
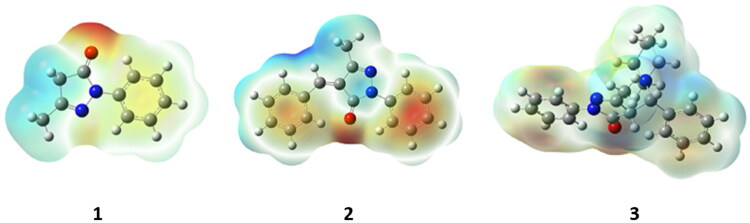
MEP surface diagrams of compounds **1–3**.

The MEP surface analysis gives the isosurfaces of nucleophilic and electrophilic attacking sites in a compound. The corresponding MEP surface diagrams of the compounds **1–3** are depicted in Figure 7. In the surface diagrams, the blue regions indicate the electropositive zones and the red zones indicate the electronegative areas in the molecules.

### Binding interaction with Tyrosinase protein

The binding propensity of compound **2** with the Tyrosinase protein (3NM8) was analysed owing to its higher antioxidant potential^89^. This comprehensive analysis provides insights into how different molecular components contribute to the binding affinity of the ligand-protein complexes, aiding in understanding their interaction dynamics and stability^90^^,^^91^.

### Molecular docking

**Figure 8.**
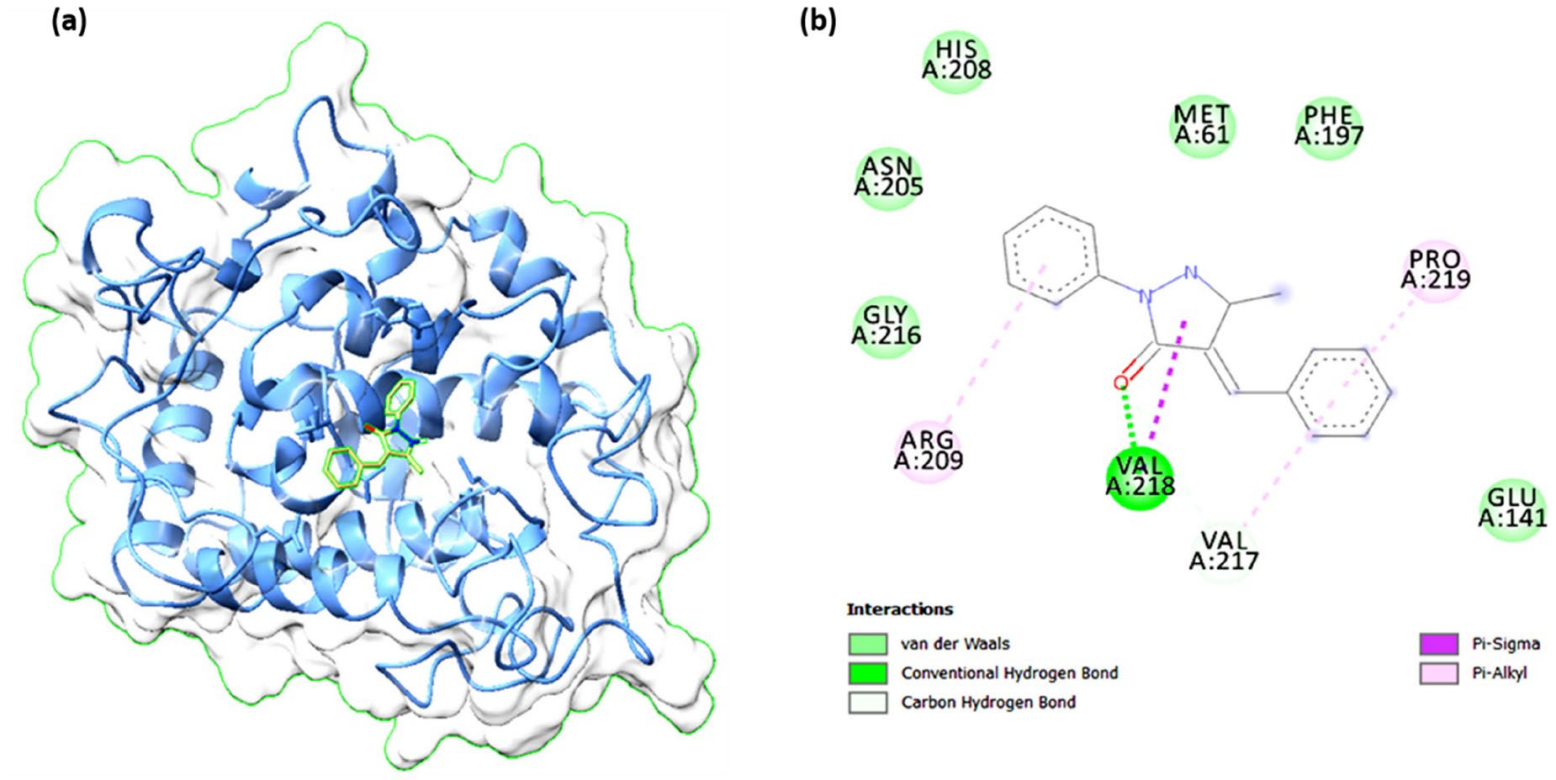
Molecular docking interaction between Tyrosinase protein (3NM8) and compound **2** with a binding affinity of −6.9 kcal/mol. (a) 3D structure showing the ligand (green) bound within the active site of 3NM8 (blue ribbon and surface representation). (b) 2D interaction diagram illustrating key binding interactions, including van der Waals (green), conventional hydrogen bonds (light green), carbon hydrogen bonds (gray), pi-alkyl (pink), and pi-sigma (purple) interactions with surrounding amino acid residues.

The molecular docking result between the protein 3NM8 and the compound **2**, with a binding affinity of −6.9 kcal/mol, indicates a moderately strong interaction, suggesting a potentially favourable binding pose within the active site of the protein (Figure 8). The 3D structure on the left side of the image shows the ribbon model of 3NM8, highlighting the ligand nestled within a binding pocket, overlaid on the protein’s surface. This positioning suggests the compound **2** is well accommodated in the hydrophobic cavity. The 2D interaction diagram on the right provides a detailed analysis of specific residue interactions with compound **2**. Key interactions include conventional hydrogen bonds between the compound **2** and VAL218, which likely stabilises the compound in its binding pose. Additionally, the presence of van der Waals interactions with multiple residues such as HIS208, ASN205, PHE197, MET61, GLU141, and others indicate proximity and compatibility between the compound **2** and the binding pocket (Figure 8). The pi-alkyl interactions with VAL217 and PRO219, and a pi-sigma interaction with VAL218, further contribute to the hydrophobic and aromatic stacking interactions that enhance the binding affinity. Carbon hydrogen bonds with residues like ARG209 and GLY216 provide additional weak stabilising forces. These collective interactions create a multi-faceted binding environment that supports the observed binding energy. The ligand orientation and interaction network reflect a good geometric and electronic complementarity to the protein’s active site, which may be crucial for any proposed inhibitory or modulatory effect. The binding energy of −6.9 kcal/mol is within a range that suggests biologically relevant binding, although further validation through experimental assays would be required. The structural complementarity and favourable interaction profile between Tyrosinase protein (3NM8) and compound **2** imply that this complex might serve as a good starting point for structure-based drug design or optimisation for therapeutic development targeting this protein.

### Molecular dynamics simulation studies

**Figure 9.**
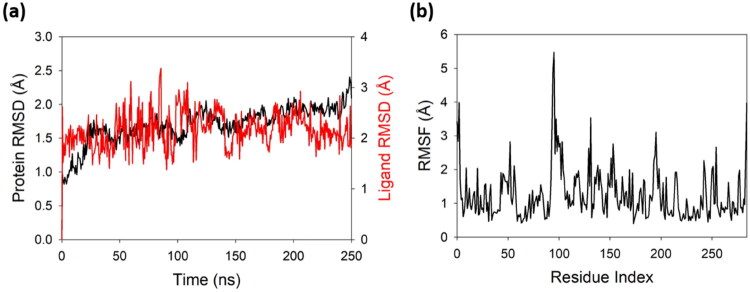
(a) RMSD plot of the 3NM8-compound 2 complex over 250 ns MD simulation. The black line represents protein backbone RMSD, while the red line shows ligand RMSD, indicating stable protein structure and sustained ligand binding throughout the trajectory (b) RMSF plot of the 3NM8-compound 2 complex over 250 ns MD simulation, showing residue-wise atomic fluctuations. Peaks indicate flexible loop or terminal regions, while low RMSF values across most residues suggest a stable protein structure and rigid binding site.

The RMSD (Root Mean Square Deviation) plot of the 3NM8 protein bound with the compound **2** offers a comprehensive view of the structural stability and conformational behaviour of the complex over a 250 ns. The protein RMSD (black line), which reflects the deviation of the protein backbone atoms from its initial conformation, shows a steady increase from ∼0.9 Å to around 1.6 Å in the first 40 ns, indicating equilibration (Figure 9(a)). From 50 ns onward, the protein RMSD fluctuates within a narrow range of 1.5–2.0 Å, reflecting a well-equilibrated and structurally stable conformation for the remainder of the simulation. Notably, there is no indication of abrupt spikes or deviations, which confirms that the overall fold of 3NM8 is preserved in the presence of compound **2**, indicating minimal disruption upon ligand binding. The ligand RMSD (red line), representing the deviation of the ligand position within the binding pocket, fluctuates more in the early stages, reaching as high as ∼2.5–2.8 Å by 100 ns, indicative of initial positional adjustments or conformational exploration. However, post-100 ns, the ligand RMSD stabilises in the 1.8–2.2 Å range, which suggests that compound **2** achieves a relatively consistent binding pose within the pocket. These sustained moderate fluctuations further suggest that the compound **2** remains bound and does not dissociate during the simulation. The close alignment of both RMSD traces over the latter half of the trajectory, with no significant divergence, supports a stable protein-molecule interaction. Taken together, the RMSD analysis demonstrates that the 3NM8-compound **2** complex is dynamically stable, with the protein maintaining structural integrity and the ligand achieving a relatively fixed, favourable conformation in the binding site. This stability under physiological conditions supports the potential bioactivity and binding relevance of compound **2** to 3NM8.

The Root Mean Square Fluctuation (RMSF) plot of the 3NM8-compound **2** complex provides a detailed residue-level analysis of atomic mobility over the course of a 250 ns molecular dynamics (MD) simulation. RMSF quantifies the average deviation of each residue’s position from its mean during the simulation, offering insight into localised flexibility and structural stability. In the plot, the y-axis represents RMSF values (in Å), while the x-axis denotes the residue index of the 3NM8 protein (Figure 9(b)). The majority of residues exhibit fluctuations below 2 Å, which is indicative of a well-structured and rigid core with minimal deviations from the average structure. Notably, several peaks can be observed, with the most prominent occurring around residue index ∼100, where RMSF reaches nearly 5.5 Å. Other moderate peaks, ranging between 2.5–4.0 Å, are distributed sporadically along the sequence. These elevated fluctuations likely correspond to surface-exposed loops, unstructured terminal ends, or flexible regions that are not tightly involved in secondary structure elements. Importantly, residues within the ligand-binding domain are expected to show lower fluctuations, suggesting that the binding of Al1 stabilises these key residues and limits their mobility, a desirable feature for strong protein-ligand interactions. The lower fluctuation values in these regions support the idea of a stable complex, where local mobility is confined mostly to peripheral or non-functional parts of the protein. Overall, the RMSF profile suggests that the 3NM8 protein maintains its structural integrity throughout the simulation, with only minor localised fluctuations. This structural rigidity, particularly around the core and the ligand-interacting residues, further supports the stable binding observed in RMSD analyses. In conclusion, the 3NM8-compound **2** complex exhibits favourable dynamic behaviour characterised by stable residue flexibility patterns, essential for the biological relevance and functional reliability of the complex.

**Figure 10.**
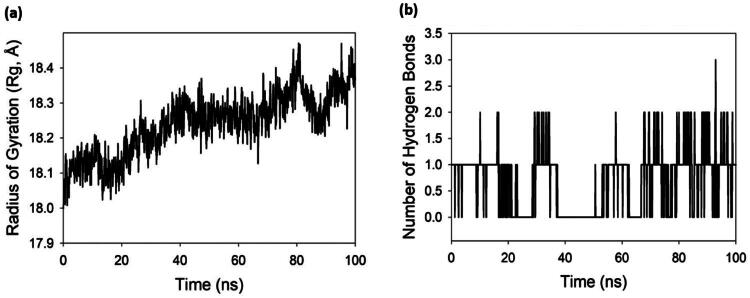
(a) Radius of gyration (Rg) plot of 3NM8 protein bound with compound **2** over 100 ns MD simulation. (b) Number of hydrogen bonds formed between 3NM8 and compound **2**.

The Radius of Gyration (Rg) plot for the 3NM8 protein bound with the compound **2 o**ver a 100 ns molecular dynamics (MD) simulation provides a critical measure of the protein’s compactness and structural integrity. Rg quantifies the distribution of atoms around the protein’s centre of mass, serving as an indicator of its folding state. At the beginning of the simulation, the Rg starts just below 18.1 Å and exhibits a gradual upward trend over time, stabilising around 18.3–18.4 Å towards the latter half of the simulation (Figure 10(a)). This slow, progressive increase in Rg suggests a mild expansion or relaxation of the protein structure, likely in response to ligand binding or solvent interactions. However, the change is subtle (only ∼0.3 Å overall), indicating that the protein does not undergo significant unfolding or denaturation. The observed fluctuations, though present, remain within a narrow range and lack any sharp or sustained spikes, reinforcing that no abrupt conformational collapse or loss of tertiary structure occurred. The relatively stable Rg profile throughout the trajectory implies that the protein maintains a well-folded, globular conformation with consistent compactness despite minor breathing motions—a hallmark of a biologically realistic simulation. The slight increase in Rg could reflect structural adaptability, especially in surface loops or domain interfaces, potentially facilitating ligand accommodation or allosteric effects. Overall, the Rg trajectory supports the conclusion that the 3NM8 protein remains conformationally stable and properly folded in the presence of the compound **2**, exhibiting no signs of destabilisation or disorder. The quality of the MD simulation is thus deemed high, with well-equilibrated dynamics and a realistic portrayal of protein behaviour under physiological conditions. Together with RMSD and RMSF analyses, the Rg data further validate the structural stability and reliability of the simulated 3NM8-compound **2** complex over the 100 ns timescale.

The hydrogen bond (H-bond) plot for the 3NM8 protein bound with the compound **2** over a 100 ns MD simulation illustrates the dynamic nature of intermolecular interactions stabilising the complex. The Y-axis indicates the number of H-bonds formed between the ligand and protein, fluctuating between 0 and a maximum of 3 throughout the trajectory. Initially, the complex maintains 1–2 hydrogen bonds consistently for the first ∼20 ns, suggesting early-stage stable interactions (Figure 10(b)). A brief reduction in bonding around 25–30 ns and again near 50–60 ns may reflect transient ligand repositioning or fluctuations in side chain conformations. However, the H-bonds promptly re-establish, particularly from 70 ns onward, where the ligand maintains 1–2 consistent hydrogen bonds with occasional spikes to 3, implying re-engagement with key residues in the binding site. Despite intermittent gaps, the recurring and sustained formation of H-bonds throughout the simulation indicates a moderate yet persistent interaction profile, characteristic of a stable yet dynamically adjusting protein–ligand complex. Importantly, the absence of prolonged zero-bond intervals beyond 20 ns and the recurring H-bonding events support the idea that the ligand remains bound and engaged, rather than drifting away or unbinding. Overall, this H-bond profile, though modest in quantity, reflects a well-behaved and biologically plausible interaction pattern, reinforcing the credibility of the simulation and the stability of the 3NM8–compound **2** complex.

**Figure 11.**
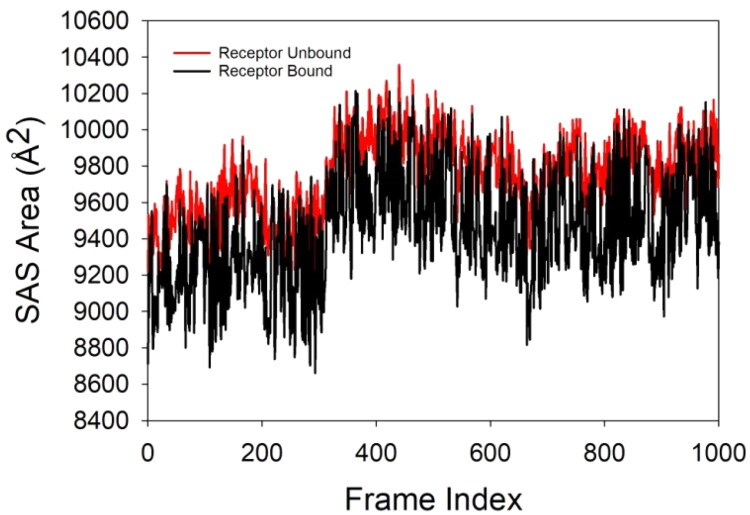
SASA plot of 3NM8 protein in unbound (red) and compound **2**-bound (black) states over 100 ns MD simulation. The bound state shows consistently lower SASA values, indicating reduced solvent exposure and enhanced structural compactness upon ligand binding.

The Solvent Accessible Surface Area (SASA) analysis of the 3NM8 protein in complex with the compound **2** over a 100 ns molecular dynamics (MD) simulation provides crucial insights into the conformational dynamics and solvation behaviour of the protein in its bound and unbound states. SASA measures the surface area of the protein that is accessible to a solvent, typically water, and serves as an indicator of protein folding, compactness, and ligand-induced conformational changes. In the provided plot, the red trace represents the receptor (protein) in its unbound state, while the black trace shows the receptor in the bound form with the compound **2** (Figure 11). It is evident that the SASA values for the bound complex are consistently lower than those of the unbound protein throughout the simulation, typically by a margin of ∼200–400 Å^2^. This reduction in SASA upon ligand binding suggests that the al1 ligand induces a more compact protein conformation or shields part of the protein surface from solvent exposure, likely by fitting snugly within a hydrophobic binding pocket. Additionally, the fluctuations in SASA are more dampened in the bound state, implying a stabilising effect of the ligand that reduces the protein’s conformational flexibility. The SASA values range approximately between 8800 and 10200 Å^2^, showing some degree of dynamic behaviour, but without any abrupt or extreme transitions, suggesting that the protein retains its globular nature and does not undergo unfolding or major destabilisation during the simulation. The consistently lower SASA in the bound state reinforces the idea of a favourable and stable interaction, corroborating the RMSD, Rg, and MMGBSA analyses. Overall, the SASA analysis supports that the binding of compound **2** to 3NM8 leads to reduced solvent exposure, structural compaction, and enhanced conformational stability of the protein, thereby validating the biological plausibility and robustness of the protein–ligand complex observed in the simulation.

The MM/GBSA (Molecular Mechanics/Generalized Born Surface Area) analysis provides a detailed thermodynamic snapshot of the binding free energy (ΔG_bind) of the 3NM8 protein with the compound **2**, with a total ΔG_bind value of −85.91 kcal/mol, suggesting a highly favourable and stable interaction. This overall negative binding free energy arises from the interplay of several contributing energetic components. The dominant stabilising forces are the van der Waals interactions (ΔG_bindvdW = −64.98 kcal/mol), which indicate that the ligand snugly fits into the protein’s hydrophobic pocket, allowing for extensive nonpolar surface contact. Complementing this is the lipophilic interaction energy (ΔG_bindLipo = −41.23 kcal/mol), further highlighting that hydrophobic forces play a crucial role in the binding affinity, possibly due to ligand burial in a largely nonpolar environment. Surprisingly, the electrostatic contributions are minimal, with the Coulombic energy (ΔG_bindCoulomb = −0.22 kcal/mol) and hydrogen bonding energy (ΔG_bindHbond = −0.16 kcal/mol) showing negligible stabilisation, suggesting that polar or charged interactions are not the primary drivers of this complex’s formation. Conversely, the solvation energy (ΔG_bindSolvGB = 23.65 kcal/mol) contributes unfavourably, as desolvation of the polar groups on both protein and ligand upon binding leads to an energetic penalty—common when hydrophobic binding dominates. The small positive covalent energy term (ΔG_bindCovalent = 2.85 kcal/mol) may represent internal strain or conformational adjustments upon binding but is not significant enough to destabilise the complex. Overall, the MM/GBSA decomposition clearly illustrates that the binding of compound **2** to 3NM8 is largely driven by hydrophobic and van der Waals forces, while electrostatic and polar interactions play a minimal role. The strong negative ΔG_bind confirms that the ligand binds favourably and stably, reinforcing its potential biological relevance and validating the robustness of the MD simulation used for energy estimation^92^^,^^93^.

## Conclusion

This research is concentrated on the evaluation of the free radical scavenging, anti-inflammatory and melanogenic inhibiting activity of the edaravone derivatives. The free radical scavenging activity of edaravone derivatives was analysed using HPSA, DPPH and ABTS assay the results were correlated. The results indicated that among the three compounds tested, compound **2**, which is a phenylethane derivative of the edaravone, shows higher antioxidant activity. In the albumin denaturation inhibition studies, compound **3** is found to exhibit marginally better activity than compound **2**, however, the anti-inflammatory activity of both the compounds were significantly lower than the standard, ibuprofen. From both the DFT analysis and Tauc plot, compound **2** is found to have low band gap values which can be correlated to its high antioxidant activity. Compound **2** also has high electrophilicity index and has exhibited higher binding energy with the Tyrosinase protein than the other two compounds. The detailed MD simulation studies also support the stability of the protein-drug complex and its potential melanogenesis inhibition property.

These results indicate that, edaravone derivatives are promising candidates for developing multifunctional drug agents against oxidative stress related conditions. Their potential can be extended from skin-related disorders such as hyperpigmentation to more complex neurodegenerative disorders, where the oxidative damage is the main contributor. Further, there is plenty of room to fine-tune these molecules to further strengthen their anti-inflammatory effect without weakening their antioxidant efficiency. This can be achieved with the help of computational tools and systematic structure–activity studies. On the other hand, it is also important to explore how these derivatives work in vivo using animal models to gain a better understanding of their mechanism of action and potential side effects. Additionally, the stability, solubility, and bioavailability of these derivatives will govern their potential for site-specific delivery, thereby determining whether they can be translated into effective pharmaceutical or cosmeceutical agents.

## Supplementary Material

SI_Final.docx

## Data Availability

The original contributions presented in the study are included in the article/Supplementary Materials, further inquiries can be directed to the corresponding author/s.
