# Supplementary material for: Evaluation of antioxidant and anti-inflammatory potential and in silico tyrosinase binding interactions of edaravone derivatives
Source: J Enzyme Inhib Med Chem. 2025 Oct 10;40(1):2561678. doi: 10.1080/14756366.2025.2561678 (PMC12517414; doi:10.1080/14756366.2025.2561678)

**Supporting Information**

**Evaluation of Antioxidant and Anti-inflammatory Potential and In Silico Tyrosinase Binding Interactions of Edaravone Derivatives**

| **Crystal analysis data of compound 2** (CCDC deposition no. 2448826).  **Table 1 Crystal data and structure refinement for al1.** | |
| --- | --- |
| Identification code | al1 |
| Empirical formula | C_17_H_14_N_2_O |
| Formula weight | 262.33 |
| Temperature/K | 296(2) |
| Crystal system | monoclinic |
| Space group | P2_1_/n |
| a/Å | 6.0544(6) |
| b/Å | 8.8927(9) |
| c/Å | 24.979(3) |
| α/° | 90 |
| β/° | 96.507(4) |
| γ/° | 90 |
| Volume/Å^3^ | 1336.2(2) |
| Z | 4 |
| ρ_calc_g/cm^3^ | 1.304 |
| μ/mm^‑1^ | 0.083 |
| F(000) | 552.0 |
| Crystal size/mm^3^ | 0.15 × 0.095 × 0.058 |
| Radiation | MoKα (λ = 0.71073) |
| 2Θ range for data collection/° | 4.866 to 50.698 |
| Index ranges | -7 ≤ h ≤ 7, -10 ≤ k ≤ 10, -30 ≤ l ≤ 30 |
| Reflections collected | 17691 |
| Independent reflections | 2441 [R_int_ = 0.0515, R_sigma_ = 0.0393] |
| Data/restraints/parameters | 2441/0/237 |
| Goodness-of-fit on F^2^ | 1.027 |
| Final R indexes [I>=2σ (I)] | R_1_ = 0.0415, wR_2_ = 0.0857 |
| Final R indexes [all data] | R_1_ = 0.0819, wR_2_ = 0.1040 |
| Largest diff. peak/hole / e Å^-3^ | 0.15/-0.15 |

| **Table 2 Fractional Atomic Coordinates (×10^4^) and Equivalent Isotropic Displacement Parameters (Å^2^×10^3^) for al1. U_eq_ is defined as 1/3 of the trace of the orthogonalised U_IJ_ tensor.** | | | | |
| --- | --- | --- | --- | --- |
| **Atom** | ***x*** | ***y*** | ***z*** | **U(eq)** |
| O1 | 7201(2) | 4117.0(17) | 5783.6(5) | 62.6(4) |
| N1 | 2783(2) | 2467.5(18) | 6347.7(6) | 49.4(4) |
| N2 | 4709(2) | 3343.5(18) | 6366.7(6) | 46.3(4) |
| C1 | 5326(3) | 2437(2) | 4561.8(8) | 45.0(5) |
| C2 | 7248(3) | 3309(2) | 4616.8(9) | 52.2(5) |
| C3 | 8453(4) | 3505(3) | 4184.6(9) | 58.9(6) |
| C4 | 7767(4) | 2852(3) | 3696.0(10) | 60.5(6) |
| C5 | 5858(4) | 2012(3) | 3630.6(9) | 61.2(6) |
| C6 | 4662(4) | 1805(3) | 4060.0(8) | 54.3(6) |
| C7 | 3941(3) | 2110(2) | 4989.2(8) | 46.7(5) |
| C8 | 4004(3) | 2475(2) | 5514.7(8) | 43.6(5) |
| C9 | 2383(3) | 1964(2) | 5856.3(8) | 44.1(5) |
| C10 | 5544(3) | 3417(2) | 5872.4(8) | 45.5(5) |
| C11 | 468(4) | 947(3) | 5709.6(10) | 53.2(6) |
| C12 | 5356(3) | 4157(2) | 6845.6(7) | 43.0(5) |
| C13 | 3895(3) | 4273(2) | 7231.6(8) | 51.3(5) |
| C14 | 4489(4) | 5100(3) | 7692.4(9) | 61.6(6) |
| C15 | 6532(4) | 5790(3) | 7773.8(10) | 64.8(6) |
| C16 | 7977(4) | 5649(3) | 7394.9(9) | 64.6(6) |
| C17 | 7424(4) | 4830(2) | 6931.1(9) | 56.1(6) |

| **Table 3 Anisotropic Displacement Parameters (Å^2^×10^3^) for al1. The Anisotropic displacement factor exponent takes the form: -2π^2^[h^2^a*^2^U_11_+2hka*b*U_12_+…].** | | | | | | |
| --- | --- | --- | --- | --- | --- | --- |
| **Atom** | **U_11_** | **U_22_** | **U_33_** | **U_23_** | **U_13_** | **U_12_** |
| O1 | 54.8(8) | 75.8(10) | 60.1(9) | -14.4(8) | 19.5(7) | -27.9(8) |
| N1 | 46.4(9) | 53.8(10) | 48.8(11) | -0.1(8) | 9.3(8) | -11.4(8) |
| N2 | 43.1(9) | 49.8(10) | 46.6(10) | -3.9(8) | 8.0(8) | -9.5(7) |
| C1 | 47.4(11) | 44.5(11) | 44.0(12) | 2.8(9) | 9.1(9) | -0.8(9) |
| C2 | 58.1(13) | 50.3(13) | 49.1(14) | 1.3(11) | 9.7(11) | -7.6(10) |
| C3 | 59.5(14) | 56.6(14) | 62.8(16) | 7.6(12) | 16.1(12) | -10.6(12) |
| C4 | 70.5(15) | 63.8(15) | 50.7(15) | 10.6(12) | 21.3(12) | 0.6(12) |
| C5 | 64.5(14) | 76.1(17) | 43.6(14) | -0.6(12) | 9.2(11) | -3.7(12) |
| C6 | 52.3(12) | 63.7(15) | 48.0(14) | -2.0(11) | 10.9(11) | -6.5(11) |
| C7 | 49.2(11) | 46.8(12) | 44.5(13) | -0.9(10) | 6.6(10) | -2.9(10) |
| C8 | 42.0(10) | 40.3(11) | 49.3(12) | 0.5(9) | 8.0(9) | -4.1(9) |
| C9 | 42.2(10) | 42.2(11) | 48.2(13) | 2.5(9) | 5.9(9) | -4.6(9) |
| C10 | 42.3(10) | 43.1(12) | 52.4(13) | -0.6(10) | 11.1(9) | -3.7(9) |
| C11 | 50.9(12) | 61.8(16) | 47.4(14) | -3.9(12) | 8.0(11) | -17.3(12) |
| C12 | 46.4(11) | 40.7(11) | 41.6(11) | -1.2(9) | 3.3(9) | 1.3(9) |
| C13 | 48.9(12) | 53.8(13) | 51.8(13) | -4.6(11) | 8.1(10) | -5.0(10) |
| C14 | 68.3(15) | 66.6(15) | 52.4(14) | -11.0(12) | 18.0(12) | -8.6(12) |
| C15 | 80.8(16) | 64.9(15) | 49.0(14) | -16.9(12) | 8.5(13) | -14.9(13) |
| C16 | 62.0(15) | 72.8(16) | 59.7(15) | -9.3(12) | 9.6(12) | -22.4(13) |
| C17 | 52.7(13) | 64.2(15) | 52.6(14) | -10.0(11) | 10.4(11) | -10.5(11) |

| **Table 4 Bond Lengths for al1.** | | | | | | |
| --- | --- | --- | --- | --- | --- | --- |
| **Atom** | **Atom** | **Length/Å** |  | **Atom** | **Atom** | **Length/Å** |
| O1 | C10 | 1.222(2) |  | C5 | C6 | 1.373(3) |
| N1 | N2 | 1.399(2) |  | C7 | C8 | 1.349(2) |
| N1 | C9 | 1.303(2) |  | C8 | C9 | 1.445(2) |
| N2 | C10 | 1.388(2) |  | C8 | C10 | 1.477(3) |
| N2 | C12 | 1.415(2) |  | C9 | C11 | 1.484(3) |
| C1 | C2 | 1.392(3) |  | C12 | C13 | 1.384(3) |
| C1 | C6 | 1.391(3) |  | C12 | C17 | 1.382(3) |
| C1 | C7 | 1.460(3) |  | C13 | C14 | 1.379(3) |
| C2 | C3 | 1.381(3) |  | C14 | C15 | 1.375(3) |
| C3 | C4 | 1.373(3) |  | C15 | C16 | 1.366(3) |
| C4 | C5 | 1.370(3) |  | C16 | C17 | 1.377(3) |

| **Table 5 Bond Angles for al1.** | | | | | | | | |
| --- | --- | --- | --- | --- | --- | --- | --- | --- |
| **Atom** | **Atom** | **Atom** | **Angle/˚** |  | **Atom** | **Atom** | **Atom** | **Angle/˚** |
| C9 | N1 | N2 | 106.77(14) |  | C9 | C8 | C10 | 104.22(16) |
| N1 | N2 | C12 | 117.62(14) |  | N1 | C9 | C8 | 112.46(16) |
| C10 | N2 | N1 | 112.49(15) |  | N1 | C9 | C11 | 119.91(17) |
| C10 | N2 | C12 | 129.36(15) |  | C8 | C9 | C11 | 127.60(18) |
| C2 | C1 | C7 | 125.58(18) |  | O1 | C10 | N2 | 125.08(18) |
| C6 | C1 | C2 | 117.86(18) |  | O1 | C10 | C8 | 130.87(18) |
| C6 | C1 | C7 | 116.55(18) |  | N2 | C10 | C8 | 104.05(15) |
| C3 | C2 | C1 | 120.2(2) |  | C13 | C12 | N2 | 119.29(17) |
| C4 | C3 | C2 | 120.6(2) |  | C17 | C12 | N2 | 120.76(17) |
| C5 | C4 | C3 | 120.2(2) |  | C17 | C12 | C13 | 119.95(19) |
| C4 | C5 | C6 | 119.5(2) |  | C14 | C13 | C12 | 119.6(2) |
| C5 | C6 | C1 | 121.7(2) |  | C15 | C14 | C13 | 120.4(2) |
| C8 | C7 | C1 | 135.13(19) |  | C16 | C15 | C14 | 119.5(2) |
| C7 | C8 | C9 | 123.57(17) |  | C15 | C16 | C17 | 121.2(2) |
| C7 | C8 | C10 | 132.20(17) |  | C16 | C17 | C12 | 119.2(2) |

| **Table 6 Hydrogen Bonds for al1.** | | | | | | |
| --- | --- | --- | --- | --- | --- | --- |
| **D** | **H** | **A** | **d(D-H)/Å** | **d(H-A)/Å** | **d(D-A)/Å** | **D-H-A/°** |
| C17 | H17 | O1 | 0.96(2) | 2.324(19) | 2.924(3) | 119.7(15) |

| **Table 7 Torsion Angles for al1.** | | | | | | | | | | |
| --- | --- | --- | --- | --- | --- | --- | --- | --- | --- | --- |
| **A** | **B** | **C** | **D** | **Angle/˚** |  | **A** | **B** | **C** | **D** | **Angle/˚** |
| N1 | N2 | C10 | O1 | 179.56(18) |  | C7 | C8 | C9 | N1 | -179.70(18) |
| N1 | N2 | C10 | C8 | -0.9(2) |  | C7 | C8 | C9 | C11 | 2.1(3) |
| N1 | N2 | C12 | C13 | -11.3(3) |  | C7 | C8 | C10 | O1 | -0.4(4) |
| N1 | N2 | C12 | C17 | 168.58(17) |  | C7 | C8 | C10 | N2 | -179.9(2) |
| N2 | N1 | C9 | C8 | -0.3(2) |  | C9 | N1 | N2 | C10 | 0.8(2) |
| N2 | N1 | C9 | C11 | 178.04(18) |  | C9 | N1 | N2 | C12 | 173.18(16) |
| N2 | C12 | C13 | C14 | -178.14(18) |  | C9 | C8 | C10 | O1 | -179.8(2) |
| N2 | C12 | C17 | C16 | 178.21(19) |  | C9 | C8 | C10 | N2 | 0.67(19) |
| C1 | C2 | C3 | C4 | 0.4(3) |  | C10 | N2 | C12 | C13 | 159.60(18) |
| C1 | C7 | C8 | C9 | -179.1(2) |  | C10 | N2 | C12 | C17 | -20.5(3) |
| C1 | C7 | C8 | C10 | 1.6(4) |  | C10 | C8 | C9 | N1 | -0.2(2) |
| C2 | C1 | C6 | C5 | 0.6(3) |  | C10 | C8 | C9 | C11 | -178.4(2) |
| C2 | C1 | C7 | C8 | -0.1(4) |  | C12 | N2 | C10 | O1 | 8.3(3) |
| C2 | C3 | C4 | C5 | 0.8(3) |  | C12 | N2 | C10 | C8 | -172.17(17) |
| C3 | C4 | C5 | C6 | -1.3(3) |  | C12 | C13 | C14 | C15 | -1.0(3) |
| C4 | C5 | C6 | C1 | 0.6(3) |  | C13 | C12 | C17 | C16 | -1.9(3) |
| C6 | C1 | C2 | C3 | -1.1(3) |  | C13 | C14 | C15 | C16 | -0.1(4) |
| C6 | C1 | C7 | C8 | 179.3(2) |  | C14 | C15 | C16 | C17 | 0.2(4) |
| C7 | C1 | C2 | C3 | 178.26(19) |  | C15 | C16 | C17 | C12 | 0.9(4) |
| C7 | C1 | C6 | C5 | -178.8(2) |  | C17 | C12 | C13 | C14 | 2.0(3) |

| **Table 8 Hydrogen Atom Coordinates (Å×10^4^) and Isotropic Displacement Parameters (Å^2^×10^3^) for al1.** | | | | |
| --- | --- | --- | --- | --- |
| **Atom** | ***x*** | ***y*** | ***z*** | **U(eq)** |
| H2 | 7740(30) | 3770(20) | 4964(9) | 72(7) |
| H3 | 9780(40) | 4070(30) | 4234(9) | 86(8) |
| H4 | 8580(30) | 2990(20) | 3401(8) | 65(6) |
| H5 | 5360(30) | 1620(20) | 3277(9) | 72(7) |
| H6 | 3290(40) | 1260(30) | 4012(9) | 80(7) |
| H7 | 2680(30) | 1440(20) | 4887(7) | 47(5) |
| H11A | -370(40) | 800(30) | 6015(10) | 92(8) |
| H11B | -530(40) | 1320(20) | 5406(9) | 79(7) |
| H11C | 970(40) | -30(30) | 5575(9) | 83(8) |
| H13 | 2450(30) | 3830(20) | 7158(7) | 57(6) |
| H14 | 3440(30) | 5220(20) | 7968(9) | 76(7) |
| H15 | 6940(30) | 6330(20) | 8084(9) | 69(7) |
| H16 | 9420(40) | 6070(20) | 7447(9) | 82(7) |
| H17 | 8470(30) | 4740(20) | 6669(8) | 66(6) |

**Check CIF report of compound 2**

**ESI-mass spectrum of Compound 3**


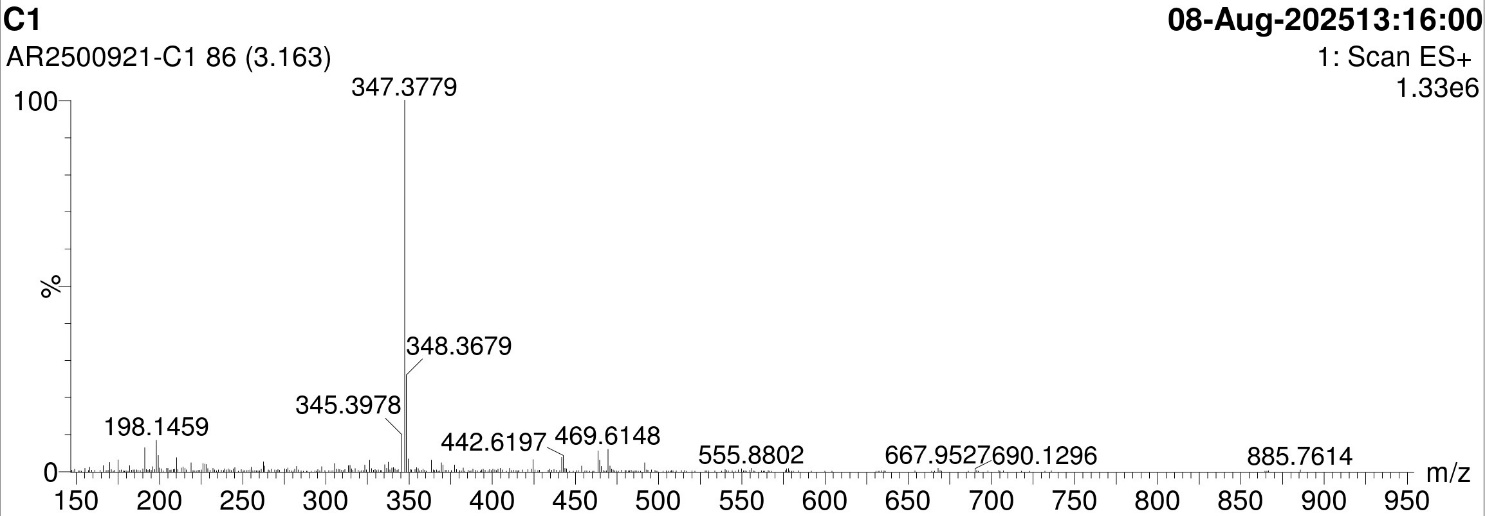

Supplement: SI_Final.docx [file IENZ_A_2561678_SM0205.docx]
